# Can you Identify This Malignancy?

**DOI:** 10.6004/jadpro.2014.5.3.9

**Published:** 2014-05-01

**Authors:** Andria N. Arrington

**Affiliations:** MD Anderson Cancer Center, Houston, Texas

## History

Mr. B. is a 39-year-old male with no significant medical history who began experiencing progressive abdominal girth and bloating. Ultrasound and CT of the abdomen and pelvis revealed moderate to large abdominal ascites. Ultrasound-guided paracentesis yielded 7 L of serous fluid, with cytology showing atypical mesothelial cells. Upper endoscopy and chest CT were unremarkable. Exploratory laparoscopy demonstrated a large amount of abdominal ascites and carcinomatosis involving the peritoneal surfaces, omentum, and surfaces of the left and right lobes of the liver. Biopsies of peritoneal surfaces and liver implants confirmed a diagnosis of mesothelioma, and Mr. B. was referred to surgical oncology.

## Chief Complaint

Mr. B. presents to the surgical oncology clinic with right upper quadrant pain extending to the right flank, 7/10 on the pain scale, exacerbated by lying on his right side. He has not been taking any pain medication. He reports abdominal bloating, progressive abdominal girth since paracentesis, early satiety, excessive gas with large meals, subjective weight gain, and dyspnea on exertion after climbing one flight of stairs. He denies nausea, emesis, melena, hematochezia, diarrhea, constipation, fevers, night sweats, and chills.

Mr. B. has never had a colonoscopy. He is a lifelong nonsmoker and denies any alcohol or drug use or any prior asbestos exposure to his knowledge. He works as a customer service representative at a department store and specifically denies any knowledge of occupational or recreational chemical exposure.

## Physical Examination and Diagnostic Studies

Upon physical exam, Mr. B. is a healthy-appearing male of stated age, in no apparent distress. His vital signs are normal. Abdominal exam reveals a dome-shaped abdomen with gross ascites. Normoactive bowel sounds are present with no hepatosplenomegaly. Shifting dullness and fluid wave are noted. There is mild tenderness to deep palpation of the right mid- abdomen. No stigmata of cirrhosis, jaundice, scleral icterus, or lower extremity edema are noted. Cardiac exam is unremarkable. Laboratory analysis reveals Mr. B.’s CA-125 level to be 263.3 U/mL (normal range: 0–35 U/mL). His CEA and CA 19-9 levels are normal, as are his complete blood count, liver function tests, and kidney function.

Cross-sectional imaging of the abdomen and pelvis (see Figure) show a large amount of ascitic fluid extending into the pelvis, with the omentum displaced and compressed by fluid as well as omental caking. No dominant mass is identified, and intra- abdominal and pelvic organs are unremarkable except for a hepatic cyst. Repeat ultrasound-guided paracentesis is performed for symptomatic relief, yielding 5 L of green fluid, with cytology again revealing atypical mesothelial cells. Mr. B. subsequently undergoes an exploratory laparoscopy revealing a large amount of abdominal ascites and carcinomatosis involving the peritoneal surfaces, surfaces of the left and right lobes of the liver, and the omentum. Representative biopsies of peritoneal and liver implants are consistent with mesothelioma.

**Figure 1:**
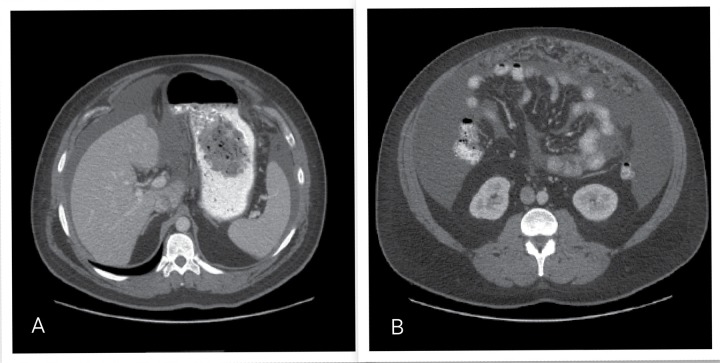
Cross-sectional imaging scans of Mr. B.’s (A) abdomen and (B) pelvis.

**Figure:**
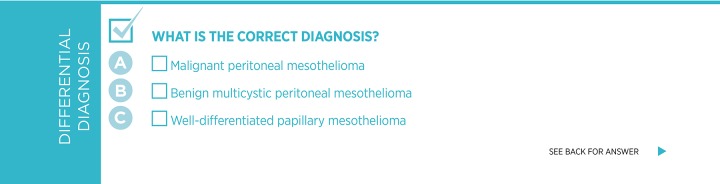
What is the correct diagnosis?

## Correct Answer: A

Malignant peritoneal mesothelioma is a rare and fatal malignancy originating from the mesothelial cells that line the peritoneum. Mr. B.’s symptomatology is characteristic of this disease, as patients typically present with a combination of ascites, abdominal pain, swelling, and early satiety (Loggie, 2001). There are approximately 400 new cases diagnosed annually in the United States, with an equal incidence in both males and females (Alexander et al., 2013). Although there has been an association of asbestos exposure with this malignancy, the cause ultimately remains unknown (Yan et al., 2009). On gross examination, there are many tumor nodules of varying size diffusely seen throughout the peritoneal cavity, often leading to massive malignant ascites. The omentum is typically infiltrated by disease; thus, radiographic evidence of omental nodularity and caking is not uncommon.

Malignant peritoneal mesothelioma should be suspected in any individual with a diffuse malignant process in the abdomen. As was the case for Mr. B., it is not uncommon for the cytology on the ascitic fluid to be negative. A definitive diagnosis can be established only through tissue biopsy with appropriate immunohistochemical staining. The major histologic variants for malignant mesothelioma are epithelioid and sarcomatoid. The epithelioid subtype accounts for approximately 75% of patients and is typically associated with better response to therapy and subsequently a better prognosis than the sarcomatoid subtype.

The role of CA-125 as a tumor marker in this malignancy is not well understood. A study assessing 46 patients undergoing cytoreductive surgery (CRS) and hyperthermic intraperitoneal chemotherapy (HIPEC) for malignant peritoneal mesothelioma reported a preoperative diagnostic sensitivity of 53.4% for CA-125 (Schaub et al., 2013). In a follow-up report, the authors described an 82% 5-year overall survival for patients with baseline CA-125 35 U/mL and a 42% 5-year overall survival for patients with CA-125 > 35 U/mL. Thus it is possible that CA-125 may parallel with disease progression and regression after CRS/HIPEC, implying that the monitoring of serial measurements in follow-up patients may be helpful.

## Explanation of Incorrect Answers

Benign multicystic peritoneal mesothelioma typically occurs in women of reproductive age (83%), is usually clinically benign, and is often discovered incidentally when a patient is having surgery for another reason (Dzieniecka & Kaluzynski, 2011). The most common presenting symptoms are chronic or intermittent abdominal or pelvic pain. Given Mr. B.’s gender and symptomatology, this is an unlikely diagnosis.

Well-differentiated papillary mesothelioma is a subtype of epithelioid mesothelioma that is less aggressive, slower growing, and typically does not metastasize to other parts of the body. Similar to benign multicystic peritoneal mesothelioma, this disease also affects women of reproductive age and is often discovered incidentally when a patient is having unrelated pelvic or abdominal surgery (Porpora et al., 2002). Patients with this condition are often asymptomatic, although chronic abdominal or pelvic pain can be present. Ascites is not typically seen with this condition. Thus, like benign multicystic peritoneal mesothelioma, papillary mesothelioma is an unlikely diagnosis for Mr. B. given his symptomatic state, especially in the presence of massive ascites.

## Management

Without treatment, the median overall survival for patients with malignant peritoneal mesothelioma is about 6 months (Alexander et al., 2013). Systemic chemotherapy using pemetrexed and cisplatin has been associated with an overall response rate of about 25% and a median overall survival of approximately 1 year. Currently, CRS with HIPEC is the treatment of choice. The goal of this type of surgery is to remove all gross disease; chemotherapy is administered intraoperatively following optimal debulking. A multi-institutional registry combining retrospective data on 405 patients with malignant peritoneal mesothelioma treated with CRS and HIPEC at 29 centers worldwide reported a median actuarial overall survival of 53 months.

## Follow-up

Mr. B. underwent CRS and HIPEC with cisplatin. His tumor was reduced to a CCR = 1 (completeness of cytoreduction), meaning that there was remaining disease that was 2.5 mm in thickness, with the bulk of the disease remaining along the interface of the mesentery to the small bowel. He was discharged from the hospital on postoperative day 8 with no postoperative complications but does suffer from minimal high-frequency hearing loss as a result of the cisplatin. Mr. B. received adjuvant carboplatin and pemetrexed for 4 cycles and is currently on maintenance pemetrexed with radiographically stable peritoneal implants.
